# Eccrine Sweat as a Biofluid for Profiling Immune Biomarkers

**DOI:** 10.1002/prca.201800010

**Published:** 2018-06-28

**Authors:** Benjamin A. Katchman, Meilin Zhu, Jennifer Blain Christen, Karen S. Anderson

**Affiliations:** ^1^ Center for Personalized Diagnostics Biodesign Institute, Arizona State University Tempe AZ 85281 USA; ^2^ School of Electrical Engineering, Arizona State University Tempe AZ 85281 USA

## Abstract

**Purpose:**

Sweat is a relatively unexplored biofluid for diagnosis and monitoring of disease states. In this study, the proteomic profiling of immune‐related biomarkers from healthy individuals are presented.

**Experimental Design:**

Eccrine sweat samples are collected from 50 healthy individuals. LC‐MS/MS is performed on two pools of sweat samples from five male and female participants. Individual sweat samples are analyzed by antibody isotyping microarrays (*n* = 49), human cytokine arrays (*n* = 30), and quantitative ELISAs for interleukin‐1α (*n* = 16), epidermal growth factor (*n* = 6), and angiogenin (*n* = 7).

**Results:**

In sweat, 220 unique proteins are identified by shotgun analysis. Detectable antibody isotypes include IgA (100% positive; median 1230 ± 28 700 pg mL^−1^), IgD (18%; 22.0 ± 119 pg mL^−1^), IgG1 (96%; 1640 ± 6750 pg mL^−1^), IgG2 (37%; 292 ± 6810 pg mL^−1^), IgG3 (71%; 74.0 ± 119 pg mL^−1^), IgG4 (69%; 43.0 ± 42.0 pg mL^−1^), and IgM (41%; 69.0 ± 1630 pg mL^−1^). Of 42 cytokines, three are readily detected in all sweat samples (*p *< 0.01). The median concentration for interleukin‐1α is 352 ± 521 pg mL^−1^, epidermal growth factor is 86.5 ± 147 pg mL^−1^, and angiogenin is 38.3 ± 96.3 pg mL^−1^. Multiple other cytokines are detected at lower levels.

**Conclusions and Clinical Relevance:**

Sweat can be used for profiling antibodies and innate immune biomarkers.

## 1 Introduction

Eccrine and apocrine glands in the epidermis produce sweat, a clear and hypotonic biofluid.^1^ Sweat is slightly acidic (pH 5.5–6.5) and is composed of mainly water, containing electrolytes (e.g., sodium, chloride, and potassium), urea, pyruvate, lactate, and peptides. In lower concentrations, antigens, antibodies, cytokines, and some xenobiotics (e.g., drugs, cosmetics, and ethanol) are also present.^2^ These substances are stored in eccrine and apocrine glands, secreted into the sweat, and transported through a sweat pore to the epidermal surface. Partial reabsorption of sodium and chloride occurs during transportation in the reabsorptive duct.^3^ Disease states can change sweat composition by altering the concentration of common components or reporting new components that may function as biomarkers for a given disease.^4^

Historically, eccrine sweat has been used to diagnose cystic fibrosis in infants and children by measuring the chloride and sodium content via pilocarpine stimulated iontophoresis. Because cystic fibrosis is the result of a defective chloride membrane transporter, reduced transport of chloride and sodium occurs in the reabsorptive duct leading to saltier sweat.^5^ Beyond electrolyte monitoring, sweat has not been commonly used as a biofluid for disease detection. Several studies have evaluated the presence of antibody isotypes and cytokines in sweat of healthy individuals. Antibody isotypes found in sweat include IgA, IgE, and IgG.^6^, ^7^, ^8^ Cytokines found in sweat include interleukin (IL)‐1α, IL‐1β, IL‐6, IL‐8, IL‐31, tumor necrosis factor (TNF)‐α, and transforming growth factor (TGF)‐β.^9^, ^10^ Additionally, studies analyzing sweat using mass spectrometry report that sweat has an abundance of apolipoprotein D, clusterin, prolactin‐inducible protein, and serum albumin.^11^

Proteomic analyses that biomarkers in sweat may correlate with certain disease states. The P.O.W.E.R. study detected elevated levels of neuropeptide Y, substance P, and calcitonin gene‐related peptide in the sweat of premenopausal women with major depressive disorder in remission.^12^ A proteomic analysis of pooled sweat samples from patients with schizophrenia showed a twofold or greater concentration of kallikrein, prostatic‐binding protein and thioredoxin compared with controls.^13^ Adewole et al. demonstrated that 26 proteins were uniquely detected in pooled sweat samples from patients with active tuberculosis.^14^ However, few studies have performed a broad, quantitative analysis of antibody isotypes and cytokines in individual sweat samples from healthy participants.

In this article, LC‐MS/MS was used to perform a global proteomic analysis on pooled sweat samples collected from 50 healthy individuals. To establish the feasibility of utilizing sweat as a source of biomarkers, we measured antibody isotypes in individual sweat samples using quantitative antibody isotyping microarrays. Further, we expanded upon previous work that demonstrated the presence of cytokines in sweat and performed a larger study probing for 42 cytokines using semi‐quantitative cytokine microarrays. Based on the results of the cytokine arrays, we selected IL‐1α, epidermal growth factor (EGF), and angiogenin (ANG) for further analysis by quantitative ELISA. This article provides quantitative data on specific antibody isotypes and cytokines present in eccrine sweat.

**Clinical Relevance**

The ability to use sweat as a biofluid provides the opportunity for noninvasive sampling for early and continuous diagnostics. To date, the contents of sweat and the mechanisms of biomarker transport from blood and interstitial fluid are relatively unexplored compared with other diagnostic biofluids. In this study, we utilized mass spectrometry, protein microarrays, and quantitative ELISAs to identify a broad range of proteins, antibody isotypes, and cytokines in sweat. The ability to detect broad spikes in immune‐related biomarkers in sweat provides researchers, clinicians, and epidemiologists a unique platform to track patients without the need for laborious hospital visits and delays in diagnosis and disease management. Since excessive sweating is a common symptom in patients with infectious disease, the identification, verification, and validation of sweat‐based immune biomarkers can lead to rapid detection and monitoring of disease states.

With recent clinical interest in continuous biomonitoring and the surge of wearable biotechnology, sweat shows promise as a biofluid providing a noninvasive source of plasma proteins, interstitial proteins, and metabolites.^15^ Due to the nominal presence of impurities, sample preparation of sweat is simpler than other biofluids. Studies have also indicated that sweat samples can also be stored for long periods of time.^16^ Biofluids, such as sweat, permit noninvasive sampling, which is critical for frequent sampling from neonates or elderly individuals and avoiding infections to patients who need daily analysis. Several laboratories have developed wearable point‐of‐care diagnostic sweat collection devices for monitoring metabolites, but currently none are able to identify protein biomarkers.^8^, ^17^, ^18^ In this article, we demonstrate the potential of eccrine sweat as a biofluid for protein detection by presenting a proteomic analysis of sweat samples from healthy individuals.

## 2 Experimental Section

*Sweat Collection*: Sixteen male and 34 female healthy individuals were recruited into the study (between 18 and 69 years of age). Informed, written consent was obtained from all participants, who were permitted to opt out at any point during the collection. Sweat samples were collected using the Macroduct wearable sweat collection device (WESCOR, Logan, UT) and the PharmChek sweat patch (PharmChem, Fortworth, TX). The volar region of each participant's forearm was first cleaned with 70% ethanol. The sweat collection devices were then fitted to the forearms following the manufacturer's instructions. Each participant was instructed to perform 30–60 min of moderate aerobic exercise indoors (stationary bike, treadmill, or elliptical). Following aerobic exercise, the sweat collection devices were removed. Sweat samples were immediately de‐identified, processed, and stored at −20 °C.

*Sample Preparation*: Samples collected via the Macroduct required liquid transfer into a microcentrifuge tube. The samples were centrifuged for 5 min at 5000 rpm in order to remove shed skin cells and cellular debris and were stored at −20 °C until further analysis. Samples collected via the PharmChek sweat patch required centrifugation to extract the liquid sample and remove shed skin cells and cellular debris. Each patch was placed in a 15 mL tube and centrifuged for 5 min at 6000 rpm. The extracted liquid sample was transferred to a microcentrifuge tube and stored at −20 °C until further analysis.

For mass spectrometry analysis, two pooled sample groups, collected using the Macroduct sweat collection device, were created by randomly selecting five samples from each the male (Group 1) and female (Group 2) individuals. The samples were pooled together to make Group 1 and Group 2. 100 μL of each liquid sample, in Group 1 and Group 2, were desalted on a 0.5 mL Zeba Desalt Column (Thermo Fisher Scientific). The protein concentration of each sample was determined using a Pierce BCA Protein Assay and 15 μg equivalent volumes were lyophilized and stored at −80 °C. All pooled samples were prepared for mass spectrometry analysis by rehydration with 200 mm Tris pH 8.2. The rehydrated samples were run on a 4–20% Criterion TGX precast gel (Bio‐Rad Laboratories, Hercules, CA) and stained with Bio‐Safe Coomossie Blue (Bio‐Rad Laboratories). Samples were then destained with 50 mm Tris pH 8.1/50% acetonitrile and reduced with 50 mm Tris(2‐carboxyethyl) phosphine hydrochloride (TCEP)/50 mm Tris pH 8.2. Alkylation of samples was performed with 50 mm Iodoacetamide/50 mm Tris pH 8.2 at room temperature in the dark for 30 min. Overnight enzymatic digestion was performed at 37 °C on 50 μL samples with 0.0025 μg μL^−1^ sequencing grade trypsin (Promega, Madison, WI) in 20 mm Tris pH 8.2. Digestion was quenched with addition of 0.2% TFA and desalting was performed with C‐18 Zip Tips (Millipore, Burlington, MA). Finally, samples were dried in a vacuum concentrator and reconstituted in 5 μL of 0.2% formic acid.

*Mass Spectrometry*: The pooled extracts were concentrated, and the proteins were identified by nano‐flow liquid chromatography electrospray tandem mass spectrometry (nano LC‐ESI‐MS/MS) using a Thermo Scientific Q‐Exactive Plus Mass Spectrometer (Thermo Fisher Scientific) coupled to a Thermo Ultimate 3000 RSLC nano HPLC system. The digested peptide mixture was loaded onto a 250 nL OPTI‐PAK trap (Optimize Technologies, Oregon City, OR) custom packed with Michrom Magic C18, 5 μm solid phase (Michrom Bioresources, Auburn, CA). Chromatography was performed using 0.2% formic acid in both the A solvent (98% water/2% acetonitrile) and B solvent (80% acetonitrile/10% isopropanol/10% water), and a 5% B to 45% B gradient over 78 min at 400 nL min^−1^ through a PicoFrit (New Objective, Woburn, MA) 100 μm × 35 cm column hand‐packed with Agilent Poroshell 120 EC C18 packing. The Q‐Exactive mass spectrometer experiment was a data‐dependent setup with a MS1 survey scan from 340–1500 *m*/*z* at resolution 70 000 (at 200 *m*/*z*), followed by HCD MS/MS scans on the top 15 ions having a charge state of +2, +3, or +4, at resolution 17 500. The ions selected for MS/MS were placed on an exclusion list for 30 s. The MS1 AGC target was set to 1.0 × 10^6^ and the MS2 target was set to 1.0 × 10^5^ with max ion inject times of 50 ms for both.

*Bioinformatics and Gene Ontology Analysis*: Tandem mass spectra were searched against the Uniprot human database, version Oct2016, using the MASCOT database search engine (Matrix Science, Boston, MA). To determine gene ontological annotations for the selected proteins, we used the Gene Ontology Consortium, PATHER Classification System were used to determine gene ontological annotations for the selected proteins.^17^

*Antibody Isotyping*: Antibody isotyping was performed on all 49 sweat samples collected via the Macroduct collection device using the Quantibody Human Ig Isotyping Array 1 (RayBiotech, Norcross, GA) according to the manufacturer's instructions with the following modifications. On day 1, each slide was brought to room temperature and allowed to dry for 1 h. Next, 100 μL of blocking buffer was added to each well and allowed to incubate for 30 min at room temperature. Sweat samples and standards were diluted in blocking buffer (10 μL of sweat into 75 μL of blocking buffer). The blocking buffer was removed from the arrays, and the diluted samples and standards were added to each well. The slides were incubated for 16 h on a rocking shaker at 4 °C. On day 2, the slides were washed according to the manufacturer's instructions. Eighty microliters of diluted biotinylated anti‐human Igs were added to each well and incubated for 16 h on a rocking shaker at 4 °C. On day 3, the slides were washed according to the manufacturer's instructions. Eighty microliters of diluted Cy3 equivalent dye‐conjugated Streptavidin was added to each well and incubated for 2 h on a rocking shaker at room temperature. Slides were washed, the gaskets removed, and the slides were dried using filtered compressed air. All slides were imaged using a Tecan Power Scanner (Tecan, Männedorf, Switzerland) and analyzed using Array‐Pro Analyzer (Meyer Instruments, Houston, TX).

Each slide contained 16 identical subarrays, in which human antibody isotypes (IgA, IgD, IgE, IgG1, IgG2, IgG3, IgG4, and IgM) were printed in quadruplicate. Prior to data analysis, each array was normalized by removing the background signal estimated by the first quartile of the nonspots and taking the log‐transforming median‐scaled raw intensities to bring the data to the same scale and stabilize the variance across the range of signals. One subarray on each slide was used to calculate the standard curve for each antibody isotype and fitted to a two‐parameter polynomial curve. For each participant, the quadruplicate spots were averaged and the standard deviation was calculated. The mean intensity values were used to determine the concentration of each antibody isotype present.

*Cytokine Arrays*: Cytokine profiling was performed using the Human Cytokine Array G‐Series 3 (RayBiotech) according to the manufacturer's instructions with the following modifications. Due to limited sample volumes, 16 sweat samples collected via the Macroduct collection device and 14 sweat samples collected via the PharmChek sweat patch were analyzed. On day 1, each slide was brought to room temperature and allowed to dry for 1 h. Next, 100 μL of blocking buffer was added to each well and allowed to incubate for 30 min at room temperature. Sweat samples were diluted in blocking buffer (15 μL of sweat into 75 μL of blocking buffer). The blocking buffer was removed from the arrays, and the diluted samples were added to each well. The slides were incubated for 16 h on a rocking shaker at 4 °C. On day 2, the slides were washed according to the manufacturer's instructions. 70 μL of diluted biotinylated anti‐cytokines was added to each well and incubated for 16 h on a rocking shaker at 4 °C. On day 3, slides were washed according to the manufacturer's instructions. 70 μL of diluted Streptavidin‐Fluor was added to each well and incubated for 16 h on a rocking shaker in the dark at 4 °C. Slides were washed, the gaskets removed, and the slides were dried using filtered compressed air. All slides were imaged using a Tecan Power Scanner and analyzed using Array‐Pro Analyzer.

Each slide contained eight identical microarrays in which 42 unique human cytokines were printed in duplicate. Prior to data analysis, each array was first normalized to six positive control spots on the first subarray. The duplicate cytokine spots and 14 negative control spots on each subarray were averaged and standard deviations calculated. Mean intensity values were considered positive if they were two standard deviations above the mean negative control intensity. An unpaired *t*‐test was performed for each cytokine and the cutoff value to identify positive cytokines.

*Quantitative ELISAs for IL‐1α, EGF, and ANG*: Sweat samples collected via the Macroduct device were selected based on sufficient sample volume and high cytokine array signals for IL‐1α (*n* = 16), EGF (*n* = 6), and ANG (*n* = 7) quantitative analysis. The Human IL‐1 alpha Platinum ELISA kit (eBioscience, San Diego, CA), Human EGF Immunoassay Kit (Invitrogen, Carlsbad, CA), and Human ANG ELISA Kit (Thermo Fisher Scientific) were used for the quantitative ELISAs. Assays were performed according to the manufacturer's instructions with the following modifications. All samples and standards were performed in duplicate. Sweat samples and standards were diluted 1:10 in the sample diluent. The standard concentrations for each quantitative ELISA ranged from 200–1 pg mL^−1^ for IL‐1α, 250–1 pg mL^−1^ for EGF, and 400–1 pg mL^−1^ for ANG. Seventy‐five microlitters of each diluted sweat sample and standard were dispensed into the microtiter plate and incubated according the manufacturer's instructions. No sample controls served as the background control, which were subtracted from the sample and standard wells. The mean intensity values of the standards were fit to a four‐parameter logistic regression to generate a standard curve. The standard curve was used to calculate the concentrations of the unknowns. All samples’ duplicate OD values were within 15% of the mean; thus, no data points were omitted from the study.

## 3 Results and Discussion

### 3.1 Identification of Proteins by Mass Spectrometry

**Figure 1:**
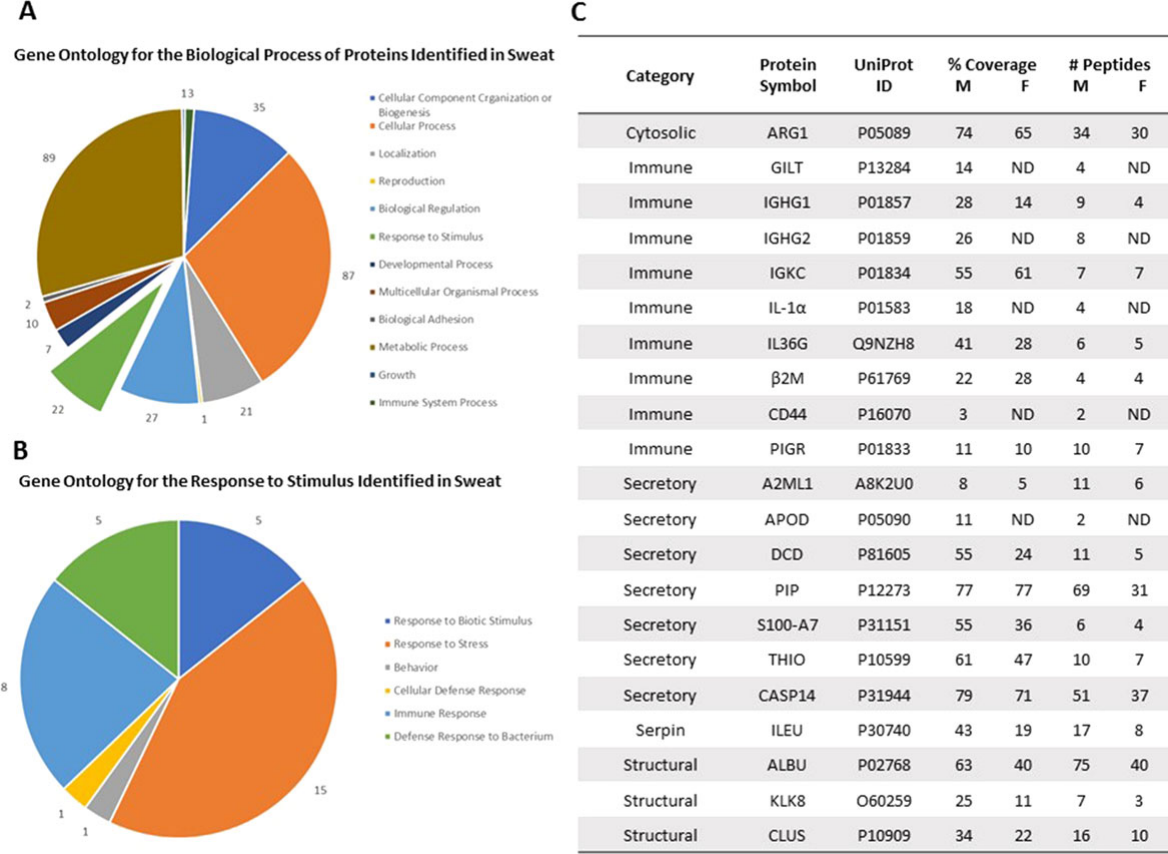
Gene ontology analysis for the biological function of proteins identified in sweat. We collected sweat from ten individuals (five males and five females) and evaluated the pooled sweat samples by LC‐MS/MS. Following LC‐MS/MS, we performed gene ontology analysis to categorize the proteins secreted in sweat based on A) biological process and B) response to stimulus. C) The sweat proteome contains a broad array of proteins, including innate and adaptive immune markers. ND means the protein was not detected.

We analyzed two pools of sweat samples from five male participants (Group 1) and five female participants (Group 2) collected via the Macroduct. Due to the limited volume of sweat and the low overall protein abundance in individual samples it was necessary to pool sweat samples for analysis. This resulted in two separate sample sets with equal total protein levels. We identified a total of 311 proteins in the male set and 189 proteins in the female set using a minimum of two peptides identified per protein (Table S1, Supporting Information). There was 93% similarity among the proteins identified between the two sample sets. This indicates a strong degree of similarity between the male and female sweat proteome, although observed minor discrepancies in the presence of certain peptides and proteins between the two groups in this limited analysis (Figure 1C).

To classify the proteins identified through LC‐MS/MS, we performed a gene ontology analysis on the combined list of identified proteins using the publicly available classification system, PANTHER (Protein Analysis Through Evolutionary Relationships). Of the 334 proteins submitted, 220 fit the criteria for classification using the PANTHER system “biological process” (Figure 1A). A comprehensive list of all the proteins identified in this study and their predicted or known biological function is presented in Table S1, Supporting Information. Similar to previous reports, we observed a high abundance of proteins involved in metabolic process (40.4%), cellular process (39.5%), and cellular component organization or biogenesis (15.9%) (Figure 1A). Within the “biological process,” 22 (10%) proteins were identified as responding to stimulus, of which the majority are involved in innate or adaptive immune responses (Figure 1).

In previous sweat proteomic studies, the proteomes of male and female sweat, both healthy and diseased, were compared to serum in an effort to understand the differences in the two biofluids.^18^ The most significant difference between serum and sweat are reported in the categories of catalytic activity, enzyme regulation, and structural molecular activity.^18^ It was also observed that there was a significantly higher amount of proteins involved in structural molecular activity. We observed a similar pattern as highlighted by glutaredoxin‐1, peroxiredoxin‐1, and quiescin sulfhydryl oxidase 1^11^, ^13^, ^14^, ^18^ (Figure 1C and Table S1, Supporting Information). In addition, the detection of membranous, cytoskeletal, microsomal, mitochondrial, ribosomal, and nuclear proteins in eccrine sweat support the hypothesis that proteins are secreted into sweat in a merocrine‐like manner, including abundant defense proteins, such as, apolipoprotein D, dermcidin, and prolactin‐inducible protein.^11^, ^13^, ^14^ Our results confirm the findings of these previous studies and support the conclusion that these collection methods provide material representative of sweat proteomes.

### 3.2 Quantitative Analysis of Antibody Isotypes

**Figure 2:**
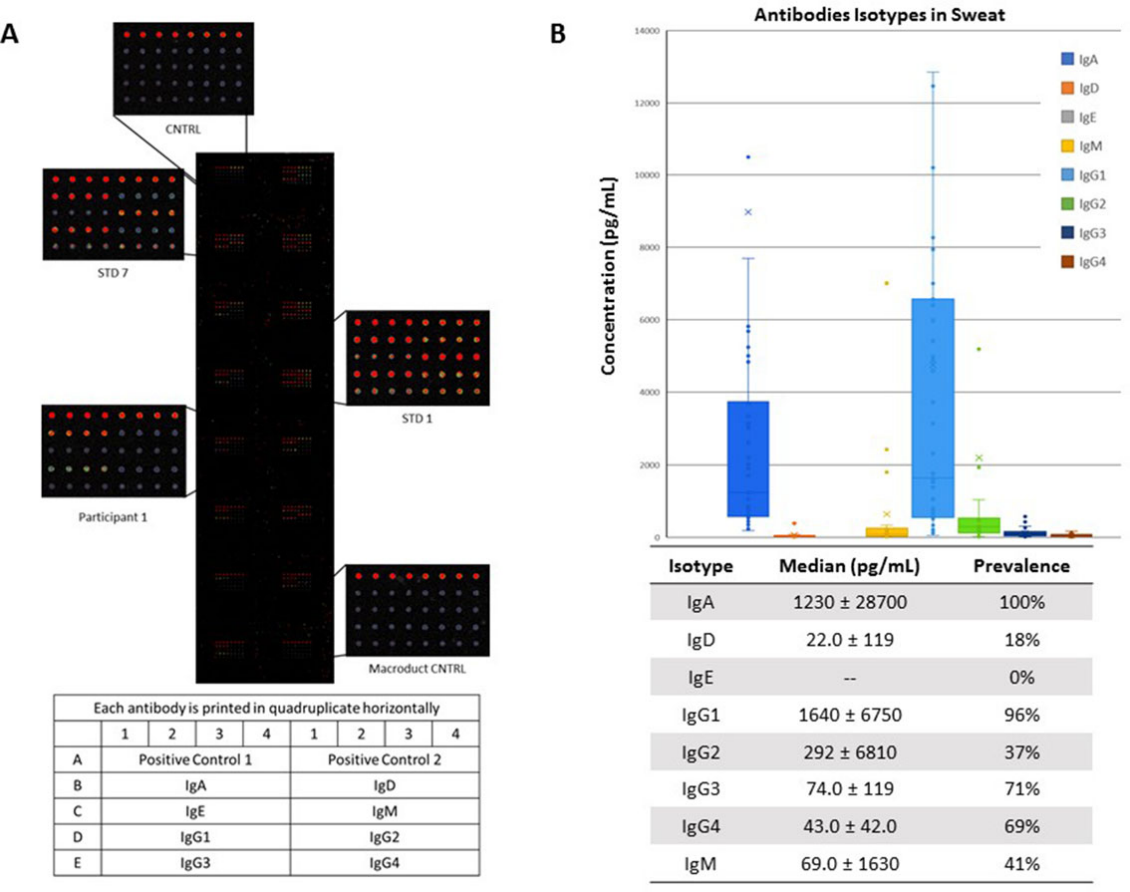
Antibody isotypes present in sweat. Sweat from 50 healthy individuals were evaluated for the concentration of human antibody (Ig) isotypes in sweat. A) An example antibody isotyping array is presented displaying the high (STD 1) and low (STD 7) concentration standards, background control sample (CNTRL), Macroduct collection device control sample (Macroduct CNTRL), and a representative participant image (Participant 1). B) The box and whisker plot represents the median, interquartile range, and range for each isotype. The table represents the median concentrations of isotypes with the standard deviations and prevalence among the participant group. The antibody isotypes quantified were IgA, IgD, IgE, IgG1, IgG2, IgG3, IgG4, and IgM.

To determine the concentration of the antibody isotypes in the eccrine sweat of 49 participants, we used human antibody isotyping arrays from RayBiotech containing IgA, IgD, IgE, IgG1, IgG2, IgG3, IgG4, and IgM (Figure 2A). All samples analyzed were collected via the Macroduct collection device. Each antibody isotype was printed in quadruplicate, and standards were run on each individual slide to determine the concentration of each antibody isotype. We detected the presence of all antibody isotypes except IgE in sweat (Figure 2B). A sample was considered positive if the mean raw value was two standard deviations higher than the mean background control (Macroduct Control, Figure 2A). The antibody isotypes IgA (100% positive; median 1232 ± 28 700 pg mL^−1^; range 37 843–1 832 631.8 pg mL^−1^) and IgG1 (96%; 1639 ± 6746 pg mL^−1^; range 14 246–380 735.5 pg mL^−1^) were present at significantly high levels in majority of individuals regardless of ethnicity, gender, or age. The concentration of antibody isotypes IgD (18%; 22 ± 119 pg mL^−1^; range 13 873–16 802 pg mL^−1^), IgM (41%; 69 ± 1627 pg mL^−1^; range 10 548.8–18 5040.5 pg mL^−1^), IgG2 (37%; 292 ± 6806 pg mL^−1^; range 13 472–30 549.8 pg mL^−1^), IgG3 (71%; 74 ± 119 pg mL^−1^; range 8646.5–15 444.3 pg mL^−1^), and IgG4 (69%; 43 ± 42 pg mL^−1^; range 10 548.8–185 040.5 pg mL^−1^) were widely variable. Our results demonstrate that all antibody isotypes, except IgE, were detectable in eccrine sweat.

Previous studies have detected IgA, IgE, and IgG antibodies secreted in sweat. These results required large volumes of sweat and have detected vastly different concentrations among men and women.^1^ Okada et al. demonstrated the presence of IgA in the sweat of healthy men (13 ± 0.9 μg mL^−1^) and women (1.6 ± 0.9 μg mL^−1^). Secreted IgA was found to be 10 times higher in men than in women, which was not observed in our study.^6^ The presence of IgE was identified in patients with atopic contact dermatitis and healthy volunteers at concentrations ranging from 1.0–75.5 ng mL^−1^.^19^ Further, one study suggested that Hepatitis B virus (HBV)‐specific IgGs were present in eccrine sweat and eccrine glands of seropositive patients.^7^ This preliminary evidence suggests that IgG and IgA levels in eccrine sweat, in particular, are sufficient to measure the seroconversion of patients with infectious diseases.

### 3.3 Identification of Human Cytokines

**Figure 3:**
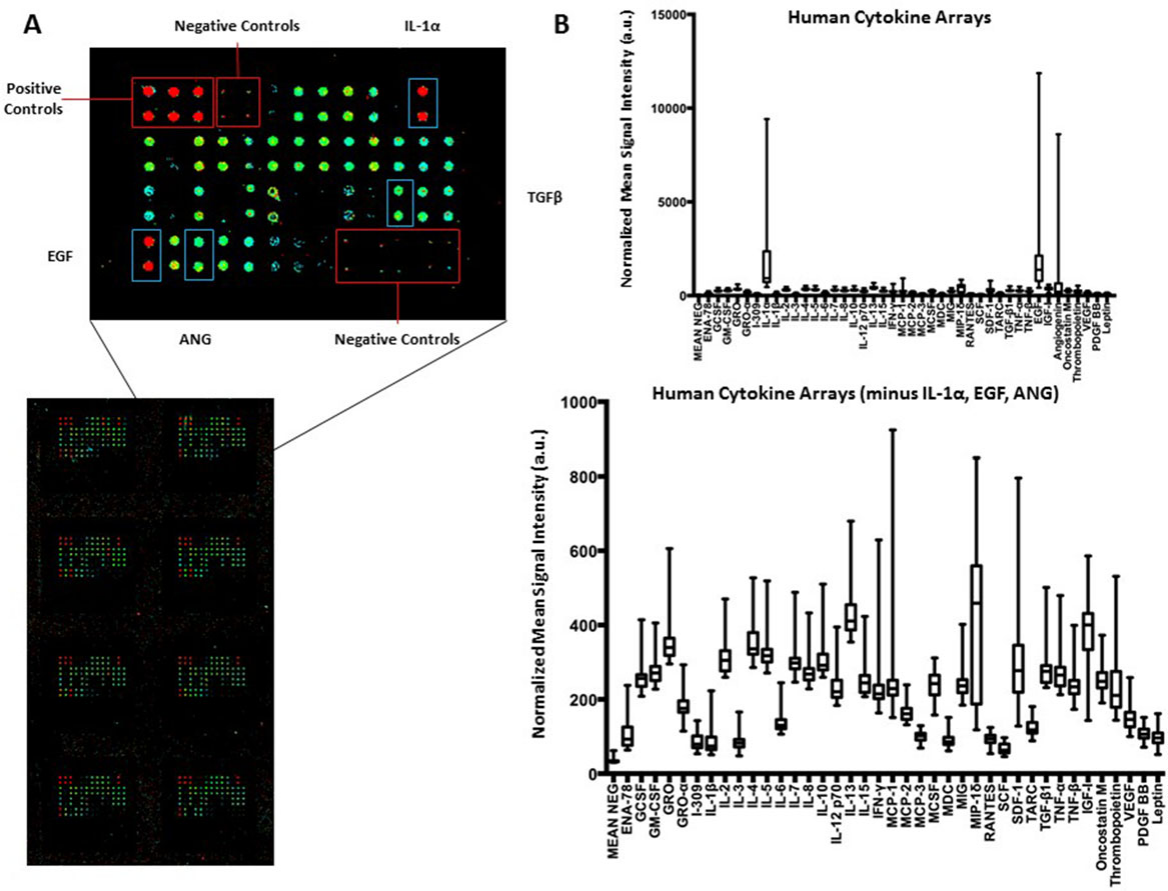
Human cytokine antibody arrays. Human cytokine arrays were used to measure the expression of 42 cytokines in 30 human sweat samples (16 Macroduct samples, 14 Pharmchek samples). A) The intensities of different cytokines (red—high, green—low) were quantified using ArrayPro Analyzer and normalized to the intensities of the internal positive controls for signal comparison. B) The normalized mean signal intensities for each cytokine and the negative control spot were plotted for each participant as a box and whisker plot representing the median, interquartile range, and range. The top graph represents all 42 cytokines and the bottom graph represent 39 of the cytokines excluding IL‐1α, EGF, and ANG.

We evaluated the presence of 42 human cytokines in 30 sweat samples using the Human Cytokine Array G‐Series 3. Of the 30 samples, 16 samples were collected via the Macroduct and 14 collected via the PharmChek. The arrays indicated whether a cytokine was present or not but not absolute concentration and 42 different human cytokines were printed in duplicate on the arrays (Figure 3A). Cytokines were considered positive if the mean signal intensity was two standard deviations above the negative control signal intensity. IL‐1α (*p* < 0.01), epidermal growth factor (*p* < 0.01), and angiogenin (*p* < 0.01) were identified as positive for all sweat samples. Other human cytokines detectable on the arrays included IL‐2, insulin‐like growth factor (IGF)‐1, IL‐13, interferon (IFN)‐γ, and macrophage inflammatory protein (MIP)‐1δ (*p* < 0.01) (Figure 3B). We compared ten of the participants’ cytokine profiles with both the Macroduct and Pharmchek (Figure S1, Supporting Information). We observed minor differences between the two sweat collection methods.

Few studies have broadly evaluated cytokines in sweat. Marques‐Deak et al. and Dai et al. performed extensive studies on identifying cytokines and immune response proteins in sweat. Marques‐Deak et al. identified IL‐1α, IL‐1β, IL‐6, IL‐8, TNF‐α, and TGF‐β in sweat samples from nine healthy women collected via a sweat patch.^10^ Dai et al. also identified IL‐1α, IL‐1β, and IL‐31 in 11 healthy volunteers collected via tissue paper.^9^ These human cytokine array results validate those findings as IL‐1α, IL‐1β, IL‐6, IL‐8, IL‐31, TNF‐α, and TGF‐β were all considered positive on the arrays. Further, our results identify additional human cytokines in sweat, including EGF, ANG, IL‐2, IGF‐1, IL‐13, IFN‐γ, monocyte chemoattractant protein (MCP)‐1, MIP‐1 δ, stromal cell‐derived factor (SDF)‐1, and IGF‐1. These results indicate that sweat contains a wider range of inflammatory and innate immune response proteins than previously observed.

### 3.4 Quantitative Analysis of IL‐1α, EGF, and ANG cytokines

**Figure 4:**
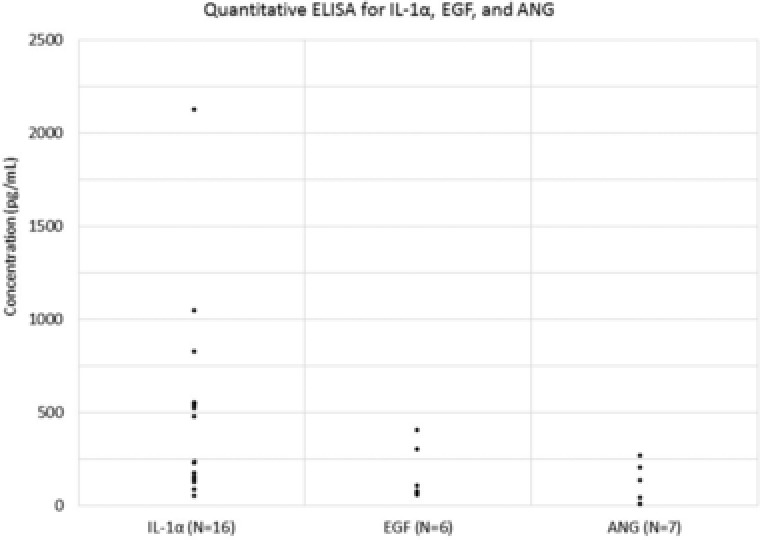
Quantitative human cytokine ELISA assay for IL1α, EGF, and ANG. Macroduct sweat samples with high signal intensities on the cytokine arrays and sufficient volumes were analyzed on quantitative ELISAs for IL‐1α, EGF, and ANG. The median concentrations were 352 ± 521 pg mL^−1^ for IL‐1α (*n* = 16), 86.5 ± 147 pg mL^−1^ for EGF (*n* = 6), and 38.3 ± 96.3 pg mL^−1^ for ANG (*n* = 7). Of the seven samples tested for ANG, three sample results were below the detectable range of the standard curve and thus, included as the LOD (1.5 pg mL^−1^) in the data summary.

To determine the concentration of IL‐1α, ANG, and EGF, participant samples with sufficient sample volume and high signal intensities on the cytokine arrays to perform on quantitative ELISAs (Figure 4). Sixteen Macroduct samples were evaluated for human IL‐1α on a quantitative ELISA. The median concentration was 352 ± 521 pg mL^−1^. IL‐1α is a cytokine that is part of the interleukin 1 family, which is responsible for producing inflammatory responses, fever, and sepsis. IL‐1α has also been investigated as a biomarker in serum for neurodegenerative disorders and alcohol abuse, and in saliva for oral lichen planus, an inflammatory condition of the oral cavity.^20^ In previous studies, Marque‐Deak et al. reported a mean concentration of 7.6 ± 3.4 pg mL^−1^ of IL‐1α in sweat samples of nine healthy participants determined by recycling immunoaffinity chromatography.^10^ In contrast, we were able to demonstrate the detection of IL‐1α in a broad range of samples using a standard ELISA.

To determine the concentration of EGF, six Macroduct samples were probed for the presence and concentration of EGF using a quantitative ELISA. The median concentration was 86.5 ± 147 pg mL^−1^. EGF stimulates cell growth and has been associated with interstitial cystitis in urine specimens.^21^ Serum EGF levels have also been found to be elevated in active psoriasis vulgaris (mean 323.0 vs 36.6 pg mL^−1^).^22^ To our knowledge, no studies have demonstrated the presence of EGF in eccrine sweat. For ANG detection, seven Macroduct sweat samples were evaluated. Three samples were below the detectable range of the standard curve and thus included as the lower limit of detection (1.5 pg mL^−1^) in the data summary. The median concentration was 38.3 ± 96.3 pg mL^−1^. Elevated ANG levels in serum have been associated with heart failure with preserved ejection fraction, bladder carcinoma, and ALS, and ANG expression in prostatic epithelial cells have been shown to increase during transition from benign to invasive prostate cancer.^23^, ^24^ To our knowledge, no studies have demonstrated the presence of ANG in sweat. ANG levels in plasma have been reported to be 308 ng mL^−1^ of 208 healthy control participants.^24^ The ability to monitor IL‐1α, EGF, and ANG in real time could provide a valuable diagnostic metric in specific disease settings.

## 4 Conclusions

Sweat is a potential diagnostic biofluid that permits noninvasive sampling. In this study, we have used mass spectrometry to perform a global proteomic analysis and microarrays to identify antibody isotypes and cytokines in eccrine sweat. Our results suggest that a broad range of IgG and IgA specific antibodies and IL‐1α, EGF, and ANG can be detected and possibly used as biomarkers in sweat for disease state monitoring. This work emphasizes that immune biomarkers can be detected and quantified in eccrine sweat, directly secreted from sweat glands or present on the skin surface. Further research is needed to evaluate the utility of these biomarkers in sweat while also taking sweat rate into account. This study provides the framework for translating these unique findings into clinical and point‐of‐care diagnostic assays.

## Conflict of Interest

Dr. Anderson serves as a consultant and member of the scientific advisory board for ProvistaDx. Dr. Katchman is currently employed by Eccrine Systems, Inc. Drs. Anderson, Katchman, and Blain Christen are co‐founders of FlexBioTech Inc.

## Supporting information

Supporting information.

Supporting information.

Supporting information.

Supporting information.
