# Supplementary figures and images for: Eccrine Sweat as a Biofluid for Profiling Immune Biomarkers
Source: Proteomics Clin Appl. 2018 Jun 28;12(6):1800010. doi: 10.1002/prca.201800010 (PMC6282813; doi:10.1002/prca.201800010)

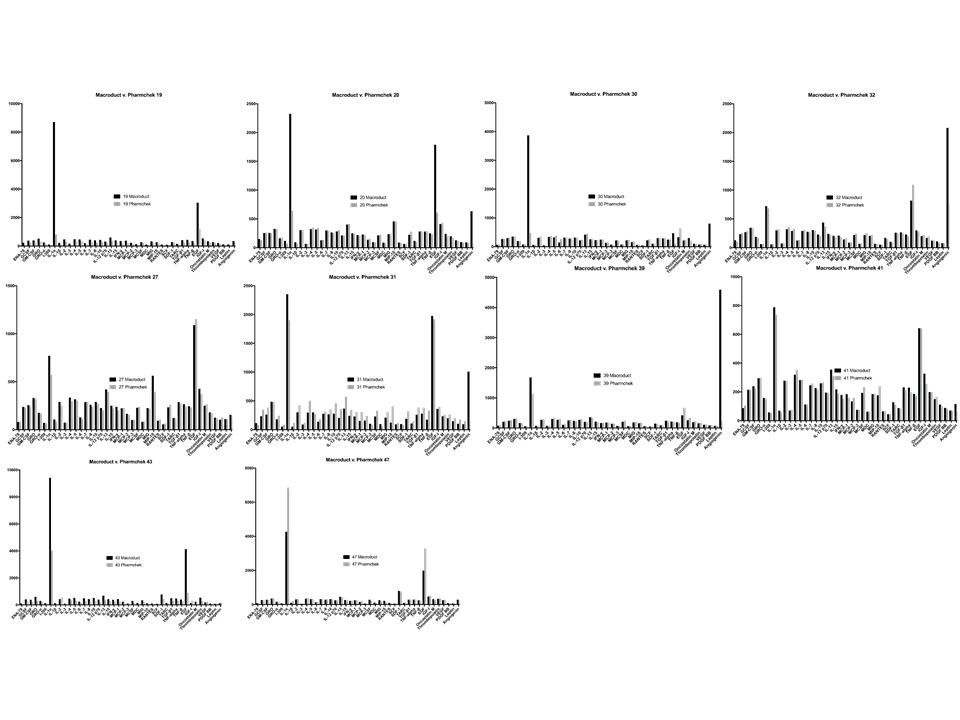

Supplement: Supplementary file 1 — Supporting information. [file PRCA-12-na-s001.jpg]
